# Molecular characterization of antimicrobial resistance and enterobacterial repetitive intergenic consensus-PCR as a molecular typing tool for *Salmonella* spp. isolated from poultry and humans

**DOI:** 10.14202/vetworld.2020.1771-1779

**Published:** 2020-09-04

**Authors:** María Paula Herrera-Sánchez, Roy Rodríguez-Hernández, Iang Schroniltgen Rondón-Barragán

**Affiliations:** 1 Research Group in Immunology and Pathogenesis, Faculty of Veterinary Medicine and Zootechnics, University of Tolima, Santa Helena Highs, Ibagué, Tolima, Colombia; 2 Poultry Research Group, Faculty of Veterinary Medicine and Zootechnics, University of Tolima, Santa Helena Highs, Ibagué, Tolima, Colombia

**Keywords:** broiler farm, genotyping, resistance genes

## Abstract

**Background and Aim:**

*Salmonella* spp. are one of the most important food-borne pathogens in the world, emerging as a major public health concern. Moreover, multidrug-resistant (MDR) strains have been isolated from salmonellosis outbreaks, which compromise its treatment success. This study was conducted to characterize the phenotypic and genotypic antibiotic resistance profile of *Salmonella* strains isolated from broilers and humans from the regions of Tolima and Santander (Colombia).

**Materials and Methods:**

*Salmonella* spp. strains (n=49) were confirmed through molecular detection by amplification of the *invA* gene. Phenotypic antibiotic resistance was determined by the automated method and the agar diffusion method, and the presence of resistance genes was evaluated by PCR. Genotypic characterization was conducted using the enterobacterial repetitive intergenic consensus (ERIC)-PCR method, from which a dendrogram was generated and the possible phylogenetic relationships were established.

**Results:**

*Salmonella* isolates were classified as MDR strains exhibiting resistance to four antibiotic classes, penicillins, aminoglycosides, sulfonamides, and cephalosporins, and the human strains were resistant to gentamicin. At the genotypic level, the isolates contained the genes *bla*_CMY2_, *bla*_CTX-M_, *bla*_PSE-1_, *bla*_TEM_, *aadA1*, *srtB*, *dfrA1*, *sul2*, and *floR*. The genotyping results obtained by ERIC-PCR allowed the grouping of strains according to the source of isolation.

**Conclusion:**

The *Salmonella* spp. strains exhibited resistance to multiple antibiotics, as well as multiple genes associated with them, and the ERIC-PCR method was a technique that was helpful in generating clusters with biological significance.

## Introduction

*Salmonella enterica* is one of the major pathogenic bacteria that can be transmitted through food consumption [1]. Consumption of products such as milk, beef, pork, chicken meat, and eggs is considered as a transmission route, based on which salmonellosis can be classified as a disease of zoonotic origin [2]. In the United States, it has been estimated that this bacterium causes 1.2 million clinical cases per year, of which 1941 outbreaks have been documented [3]. In contrast, in Colombia, the clinical cases that were reported from during 2000-2013 were most frequently caused by the serotypes Typhimurium and Enteritidis [4].

Furthermore, several serotypes of *Salmonella* spp. have been reported to be antibiotic-resistant, which represents a public health problem due to the risk of transmission of resistance between bacterial populations. Due to the plasticity of these bacteria, they have adapted and developed mechanisms to resist the effects of antibiotics using genetic strategies such as gene mutations or acquisition of resistance genes by horizontal transfer [5]. One of the primary causes of this resistance is the use of antibiotics as growth promoters in animal diets or their direct use for prophylactic purposes [6].

In *Salmonella* and other bacteria, genotyping methods have been used for identifying the clonal and phylogenetic relationships between different isolates to generate control strategies and for surveillance of outbreaks caused due to multidrug-resistant (MDR) bacteria [7]. Repetitive element-based PCR (rep-PCR) is a simple and inexpensive method that can be used to discriminate between *Salmonella* strains through the analysis of band patterns. The enterobacterial repetitive intergenic consensus (ERIC) is a repetitive sequence that is highly conserved and located in the intergenic zones; it has a variable distribution along the bacterial chromosome, separated by different lengths of intragenic sequences, which allows these primers to offer different band profiles [8,9]. The REP-PCR technique has also been widely applied in *Salmonella* studies [8,10].

Therefore, the aim of this study was to characterize the phenotypic and genotypic antibiotic resistance profile of *Salmonella* strains isolated from broilers and human subjects from the regions of Tolima and Santander (Colombia).

## Materials and Methods

### Ethical approval

No ethical approval required for this study because samples were from Bacterial Strain Collection of the Laboratory of Immunology and Molecular Biology. All the procedures for the previous collection of the samples from animals and humans were approved by Bioethics Committee of the Central Office of Research from University of Tolima and complied with the guidelines for animal care and use in research and teaching.

### Study period and location

The *Salmonella* strains from Tolima broiler farms were collected from March 2015 to March 2016. The strains from Santander broiler farms were collected from January 2015 to December 2015. In the case of the human strains, they were collected from August 2015 to December 2015 in local health care centers in Ibagué - Tolima. Finally, the study was conducted from October 2018 to June 2019 in the Laboratory of Immunology and Molecular Biology – LIBM of the University of Tolima.

### *Salmonella* spp. strains

*Salmonella* spp. strains were obtained from the previous studies conducted by the Poultry Research Group of the University of Tolima [11,12]. A total of 39 strains of *Salmonella* spp. isolated from broiler farms were included, of which 15 strains were serotyped as *Salmonella* Heidelberg (Santander broiler farms), and 24 strains were serotyped as *Salmonella* Paratyphi B (Tolima broiler farms), according to the Kauffman–White–Le Minor scheme [13]. In addition, ten strains isolated from human subjects with gastroenteritis in Tolima region were included in the study, which belonged to the serotypes Newport (n=1), Enteritidis (n=4), Braenderup (n=1), Uganda (n=1), Typhimurium (n=2), and Grupensis (n=1).

### Molecular confirmation

Genomic DNA (gDNA) was extracted from fresh colonies using the Invisorb^®^ Spin Universal Kit (Stratec, Germany) and maintained at −20°C until use. All isolates were confirmed by PCR by the amplification of the *invA* gene (accession number NC003197.2) using the primers forward 5′-TGAAATTATCGCCACGTTCGGGCAA-3′ and reverse 5′-TCATCGCACCGTCAAAGGAACC-3′ with an amplicon size of 284 bp. *S. enterica* ATCC^®^ 13076 strain (ATCC, USA) was used as a positive control. The PCR assay was conducted in a total volume of 25 μL consisting of 14.87 μL distilled deionized water, 5 μL of 5× colorless GoTaq^®^ Flexi Buffer (Promega, USA), 1 μL dNTPs (1.5 mM) (Invitrogen, USA), 1 μL of each primer (forward and reverse) (10 pmol/μL), 1 μL MgCl_2_ (25 mM), 0.125 μL of 0.6 U GoTaq^®^ Flexi DNA polymerase (Promega, USA), and 1 μL gDNA as the template. The amplification was performed in a T-100™ thermocycler (Bio-Rad, USA) with an initial denaturation step at 95°C for 3 min, followed by 35 cycles of denaturation at 95°C for 30 s, annealing at 55°C for 30 s, extension at 72°C for 30 s, and a final step of extension at 72°C for 7 min. The amplicons were visualized on 2% agarose gel by electrophoresis (PowerPac™ HC, Bio-Rad, USA) using 100-bpDNA ladder Load Ready™ (Amplyus, USA). The gel was stained with HydraGreen™ (ACTGene, USA) and visualized under the UV light using the ENDURO™ GDS gel documentation system (Labnet International, Inc., USA).

### Phenotypic resistance

The phenotypic resistance to ampicillin (AM) (4-16 μg/mL), piperacillin/tazobactam (4/4-64/4 μg/mL), gentamicin (GM) (2-8 μg/mL), trimethoprim/sulfamethoxazole (SXT) (1/19-4/76 μg/mL), ceftriaxone (CRO) (1-32 μg/mL), ceftazidime (CAZ) (1-16 μg/mL), cefepime (1-16 μg/mL), ertapenem (ETP) (0.25-4 μg/mL), imipenem (1-8 μg/mL), and meropenem (1-8 μg/mL) was evaluated using the automated BD Phoenix NMIC/ID-94 (Becton Dickinson, USA) through the minimum inhibitory concentration method following the recommendations of the CLSI [14]. The resistance to chloramphenicol (CHL, 30 μg), florfenicol (FFC, 30 μg), and streptomycin (STR, 10 μg) was determined using the Kirby–Bauer disk diffusion susceptibility test. A bacterial suspension was spread onto Mueller-Hinton agar (Oxoid, Germany), according to the McFarland turbidity scale of 0.5, and then, the bacterial growth inhibition was determined at 37°C for 18 h according to the CLSI [14] guidelines.

### Genotypic resistance

The presence of antimicrobial resistance genes was determined by PCR using gene-specific primers described in Table-1. The gDNA extracted from the isolates was used as the template for the PCR assay that was conducted under the above-described conditions, except that the annealing temperature was adjusted according to the melting temperature of each primer set.

**Table-1:** Primers used to evaluate the presence of resistance genes [42] in *Salmonella* spp. strains.

Antibiotic	Target gene	Primer sequence	Amplicon size (bp)
Ampicillin	*bla*_PSE-1_	F- GCAAGTAGGGCAGGCAATCA R- GAGCTAGATAGATGCTCACAA	461
	*bla*_TEM_	F- ATCAGTTGGGTGCACGAGTG R- ACGCTCACCGGCTCCAGA	608
Chloramphenicol	*catA*	F- CCAGACCGTTCAGCTGGATA R- CATCAGCACCTTGTCGCCT	454
	*cmlA*	F- TGGACCGCTATCGGACCG R- CGCAAGACACTTGGGCTGC	642
Florfenicol	*floR*	F- CACGTTGAGCCTCTATATGG R- ATGCAGAAGTAGAACGCGAC	888
Gentamicin	*aadB*	F-CTAGCTGCGGCAGATGAGC R-CTCAGCCGCCTCTGGGCA	300
Streptomycin	*aadA1*	F- CTCCGCAGTGGATGGCGG R- GATCTGCGCGCGAGGCCA	629
	*aadA2*	F- CATTGAGCGCCATCTGGAAT R- ACATTTCGCTCATCGCCGGC	501
	*strA*	F- TGGCAGGAGGAACAGGAGG R- AGGTCGATCAGACCCGTGC	404
	*strB*	F- GCGGACACCTTTTCCAGCCT R- TCCGCCATCTGTGCAATGCG	620
Trimethoprim	*dfrA1*	F- CAATGGCTGTTGGTTGGAC R- CCGGCTCGATGTCTATTGT	253
	*dfrA10*	F- TCAAGGCAAATTACCTTGGC R- ATCTATTGGATCACCTACCC	433
	*dfrA12*	F- TTCGCAGACTCACTGAGGG R- CGGTTGAGACAAGCTCGAAT	330
Ceftriaxone	*bla*_CMY2_	F- AAATCGTTATGCTGCGCTCT R- CCGATCCTAGCTCAAACAGC	244
	*bla*_CTX-M_	F- TTCGCTAAATACCGCCATTC R- TATCGTTGGTTGTGCCGTAA	236
Sulfamethoxazole	*sul1*	F- CGGACGCGAGGCCTGTATC R- GGGTGCGGACGTAGTCAGC	591
	*sul2*	F- GCGCAGGCGCGTAAGCTGAT R- CGAAGCGCAGCCGCAATTC	514
	*sul3*	F- GGGAGCCGCTTCCAGTAAT R- TCCGTGACACTGCAATCATTA	500

### ERIC-PCR

The 49 *Salmonella* spp. strains were fingerprinted using the primer set ERIC1 5′-ATGTAAGCTCCTGGGGATTCA-3′ and ERIC2 5′-AAGTAAGTGACTGGGGTGAGAGCG-3′ [9]. The PCR was performed in a total volume of 25 μL containing 11.85 μL distilled deionized water, 5 μL of 5× colorless GoTaq^®^ Flexi Buffer (Promega, USA), 2 μL dNTPs (1.5 mM) (Invitrogen, USA), 1 μL of each primer (50 pmol/μL), 2 μL MgCl_2_ (25 mM), 0.15 μL of 0.7 U GoTaq^®^ Flexi DNA polymerase (Promega, USA), and 2 μL gDNA as the template. The amplification was conducted in a T-100™ thermocycler (Bio-Rad, USA) with an initial denaturation step at 94°C for 5 min, followed by 35 cycles of denaturation at 94°C for 30 s, annealing at 52°C for 1 min, extension at 72°C for 7 min, and a final step of extension at 72°C for 10 min.

The PCR products were visualized by horizontal electrophoresis using 1% agarose gel (UltraPure™ Agarose, Invitrogen, USA) in 0.5× TBE, and the gel was stained with HydraGreen™ (ACTGene, Piscataway, USA). A 1-kb DNA ladder (Solis BioDyne, Estonia) was used in each gel as a molecular weight marker. The PCR products were run at 50 V for 3 h 30 min. For the cluster analysis, the banding patterns were analyzed using the BioNumerics version 7.5 software (Applied Maths, Sint-Martens-Latem, Belgium), and the genetic distances between strains were calculated according to the dice coefficient [15]. The dendrogram was constructed using the unweighted pair group method with arithmetic mean (UPGMA) in the BioNumerics version 7.5 software (Applied Maths, Sint-Martens-Latem, Belgium). In addition, the discriminatory index was calculated using the formula described by Hunter and Gaston [16] based on Simpson’s diversity index.

## Results

### Molecular confirmation

In all the 49 strains, the 284-bp fragment of the gene *invA* could be amplified, which indicated all the strains belonged to the genus *Salmonella*.

### Phenotypic resistance

The 39 *Salmonella* spp. strains isolated from broiler farms were classified as MDR strains that were resistant to the four antibiotic classes of penicillins, aminoglycosides, sulfonamides, and cephalosporins (AM, GM, STR, SXT, CRO, and CAZ) (Table-2). Regarding the strains isolated from human subjects with gastroenteritis, the serotype Typhimurium (n=1) was classified as an MDR strain that exhibited resistance to GM, STR, chloramphenicol, and florfenicol. In total, 83.6% (41/49) of the strains were resistant to STR and 79.5% (39/49) were resistant to AM. In the case of cephalosporins, 75.5% (37/49) of the strains were resistant to CRO and CAZ. In addition, 71.4% (35/49) of the strains were resistant to SXT, and 65.3% (32/49) were resistant to GM. In contrast, all (100%; 49/49) the strains were susceptible to ETP, and 97.9% (48/49) of the strains were susceptible to amphenicols and carbapenems.

**Table-2:** Phenotypic and genotypic profiles of resistance in *Salmonella* spp. strains.

Strain code	Phenotypic antimicrobial resistance profile	Genotypic antimicrobial resistance profile
1	AM, STR, SXT, CRO, CAZ	*aadA1, strA, strB, sul1, sul2, bla*_CMY2_
2	AM, STR, SXT, CRO, CAZ	*strA, strB, sul1, sul2, bla*_CMY2_
3	AM, STR, SXT, CRO, CAZ	*aadA1, strA, strB, sul1, sul2, bla*_CMY2_
4	AM, STR, SXT, CRO, CAZ	*strA, strB, sul1, sul2, bla*_CMY2_
5	AM, STR, SXT, CRO, CAZ	*aadA1, strA, strB, sul1, bla*_CMY2_
6	AM, STR, SXT, CRO, CAZ	*aadA1, strA, strB, sul1, sul2, bla*_CMY2_
7	AM, STR, SXT, CRO, CAZ	*strA, strB, sul1, bla*_CMY2_
8	AM, STR, SXT, CRO, CAZ	*strA, strB, sul1, sul2, bla*_CMY2_
9	AM, STR, SXT, CRO, CAZ, FEP	*aadA1, strA, strB, sul1, sul2, bla*_CMY2_
10	AM, STR, SXT, CRO, CAZ, FEP	*aadA1, strA, strB, sul1, sul2, bla*_CMY2_
11	AM, STR, CRO, CAZ, FEP	*aadA1, strA, strB, sul1, sul2, bla*_CMY2_
12	AM, TZP, STR, SXT, CRO, CAZ, FEP	*aadA1, strA, strB, sul1, sul2, bla*_CMY2_
13	AM, STR, SXT, CRO, CAZ, FEP	*strA, strB, sul1, sul2, bla*_CMY2_
14	AM, STR, SXT, CRO, CAZ, FEP	*aadA1, strA, strB, sul1, sul2, bla*_CMY2_
15	AM, STR, SXT, CRO, CAZ, FEP	*strA, strB, sul1, sul2, bla*_CMY2_
16	AM, GM, STR	*bla*_PSE-1_*, bla*_TEM_*, aadA1, dfrA1, bla*_CMY2_
17	AM, GM, STR, CRO, CAZ	*bla*_PSE-1_*, bla*_TEM_*, aadA1, strB, dfrA1, sul2, bla*_CMY2_
18	AM, GM, STR, SXT, CRO, CAZ	*bla*_PSE-1_*, bla*_TEM_*, aadA1, strB, dfrA1, sul2, bla*_CMY2_
19	AM, STR, CRO, CAZ	*bla*_PSE-1_*, bla*_TEM_*, aadA1, strB, dfrA1, sul1, sul2, bla*_CMY2_
20	AM, GM, STR, CRO, CAZ	*bla*_PSE-1_, *bla*_TEM_, *aadA1, strA, dfrA1, sul2, bla*_CMY2_
21	AM, GM, STR, SXT, CRO, CAZ	*bla*_PSE-1_*, bla*_TEM_*, aadA1, dfrA1, sul2, bla*_CMY2_, *bla*_CTX-M_
22	AM, STR, SXT, CRO, CAZ	*bla*_PSE-1_*, bla*_TEM_*, aadA1, strA, dfrA1, sul2, bla*_CMY2_*, bla*_CTX-M_
23	AM, GM, STR, SXT, CRO, CAZ	*bla*_PSE-1_, *bla*_TEM_*, aadA1, dfrA1, sul1, sul2, bla*_CMY2_
24	AM, GM, STR, SXT, CRO, CAZ	*bla*_PSE-1_*, bla*_TEM_*, aadA1, dfrA1, strA, strB, bla*_CMY2_
25	AM, GM, STR, SXT, CRO, CAZ	*bla*_PSE-1_*, bla*_TEM_*, aadA1, strA, strB, dfrA1, sul1, sul2, bla*_CMY2_*, bla*_CTX-M_
26	AM, GM, STR, SXT, CRO, CAZ, IPM	*bla*_PSE-1_*, bla*_TEM_, *aadA1, strA, strB, dfrA1, sul1, sul2, bla*_CMY2_*, bla*_CTX-M_
27	AM, GM, STR, SXT, CRO, CAZ	*bla*_PSE-1_*, bla*_TEM_*, aadA1, strA, strB, dfrA1, sul1, sul2, bla*_CMY2_*, bla*_CTX-M_
28	GM, STR, SXT	*bla*_PSE-1_*, bla*_TEM_, *aadA1, strA, strB, dfrA1, sul1, sul2, bla*_CMY2_
29	AM, GM, STR, SXT, CRO, CAZ	*bla*_PSE-1_*, bla*_TEM_*, aadA1, strB, dfrA1, sul2, bla*_CMY2_
30	AM, GM, STR, SXT, CRO, CAZ	*bla*_PSE-1_*, bla*_TEM_,* aadA1, strA, strB, dfrA1, sul1, sul2, bla*_CMY2_
31	AM, GM, STR, SXT, CRO, CAZ	*bla*_PSE-1_*, bla*_TEM_*, aadA1, strA, strB, dfrA1, sul1, sul2, bla*_CMY2_
32	AM, GM, STR, SXT, CRO, CAZ	*bla*_PSE-1_*, bla*_TEM_, *aadA1, strA, strB, dfrA1, sul1, sul2, bla*_CMY2_*, bla*_CTX-M_
33	AM, GM, STR, SXT, CRO, CAZ	*bla*_PSE-1_*, bla*_TEM_*, aadA1, strA, strB, dfrA1, sul1, sul2, bla*_CMY2_
34	AM, GM, STR, SXT, CRO, CAZ	*bla*_PSE-1_*, bla*_TEM_, *aadA1, strA, strB, dfrA1, sul1, sul2, bla*_CMY2_*, bla*_CTX-M_
35	AM, GM, STR, SXT, CRO, CAZ	*bla*_PSE-1_*, bla*_TEM_*, aadA1, strA, strB, dfrA1, sul1, sul2, bla*_CMY2_*, bla*_CTX-M_
36	AM, GM, STR, SXT, CRO, CAZ	*bla*_PSE-1_*, bla*_TEM_*, aadA1, aadA2, strA, strB, dfrA1, dfrA12, sul2, bla*_CMY2_
37	AM, GM, STR, SXT, CRO, CAZ	*bla*_PSE-1_*, bla*_TEM_*, aadA1, aadA2, strA, strB, dfrA1, dfrA12, sul2, cmlA, bla*_CMY2_
38	AM, GM, STR, SXT, CRO, CAZ	*bla*_PSE-1_*, bla*_TEM_*, aadA1, aadA2, strA, strB, dfrA1, sul2, bla*_CMY2_
39	AM, GM, STR, SXT, CRO, CAZ	*bla*_PSE-1_*, bla*_TEM_, *aadA1, strA, strB, dfrA1, dfrA12, sul2, bla*_CMY2_
40	GM	*bla*_PSE-1_*, bla*_TEM_*, aadA1, strB, dfrA1, dfrA12, sul2, bla*_CMY2_
41	GM	*bla*_PSE-1_*, bla*_TEM_*, aadA1, aadA2, strA, strB, dfrA1, dfrA12, sul2, bla*_CMY2_
42	GM	*bla*_PSE-1_*, bla*_TEM_*, aadA1, aadA2, strB, dfrA1, dfrA12, sul2, bla*_CMY2_
43	GM	*bla*_PSE-1_*, bla*_TEM_*, aadA1, strA, strB, dfrA1, dfrA12, sul2, bla*_CMY2_
44	GM	*bla*_PSE-1_*, bla*_TEM_*, aadA1, aadA2, strB, dfrA1, dfrA12, sul2, bla*_CMY2_
45	GM	*bla*_PSE-1_*, bla*_TEM_*, aadA1, aadA2, strA, strB, dfrA1, dfrA12, sul2, bla*_CMY2_
46	GM	*bla*_PSE-1_*, bla*_TEM_*, aadA1, aadA2, strB, dfrA1, dfrA12, sul2, bla*_CMY2_
47	AM, GM, SXT	*bla*_PSE-1_*, bla*_TEM_*, aadA1, aadA2, strB, dfrA1, dfrA12, sul2, sul3, cmlA, bla*_CMY2_
48	GM	*bla*_PSE-1_*, bla*_TEM_*, aadA1, aadA2, strB, dfrA1, dfrA12, sul2, cmlA, bla*_CMY2_
49	GM, STR, CHL, FFC	*bla*_PSE-1_*, bla*_TEM_*, aadA1, aadA2, strB, dfrA1, dfrA12, sul2, bla*_CMY2_

AM=Ampicillin, TZP=Piperacillin/tazobactam, GM=Gentamicin, STR=Streptomycin, SXT=Trimethoprim/sulfamethoxazole, CHL=Chloramphenicol, FFC=Florfenicol, CRO=Ceftriaxone, CAZ=Ceftazidime, FEP=Cefepime, IPM=Imipenem. Poultry farms (Santander): Heidelberg (1-15); Poultry farms (Tolima): Paratyphi B (16-39); Human: Newport (40), Enteritidis (41-43), Braenderup (44), Uganda (45), Enteritidis (46), Typhimurium (47), Grupensis (48), Typhimurium (49)

### Genotypic resistance

The gene *bla*_CMY2_ that confers resistance to CRO was detected in all the strains; however, some *Salmonella* spp. strains isolated from human subjects did not exhibit phenotypic resistance to this antibiotic (Table-2). Furthermore, in 93.9% of the strains that demonstrated the *sul2* gene and in 69.4% of the strains showing the *dfrA1* gene, these two genes conferred resistance to SXT. Moreover, other genes (*sul1*, *sul3*, *dfrA10*, and *dfrA12*) were evaluated, and it was observed that some strains contained one to three genes that can confer resistance to this antibiotic. However, some strains isolated from human subjects did not demonstrate phenotypic resistance. In addition, 69.4% of the strains demonstrated the presence of the genes *bla*_PSE-1_ and *bla*_TEM_ that conferred resistance to AM, and 87.8% of the strains showed the presence of the genes *aadA1* and *strB* that conferred resistance to STR. The gene *aadB* was not detected in the strains; however, the *Salmonella* spp. strains isolated from human subjects were phenotypically resistant to GM. Furthermore, the *S*. Typhimurium strain that was phenotypically resistant to amphenicols demonstrated the presence of the gene *floR*.

### ERIC-PCR

**Figure-1:**
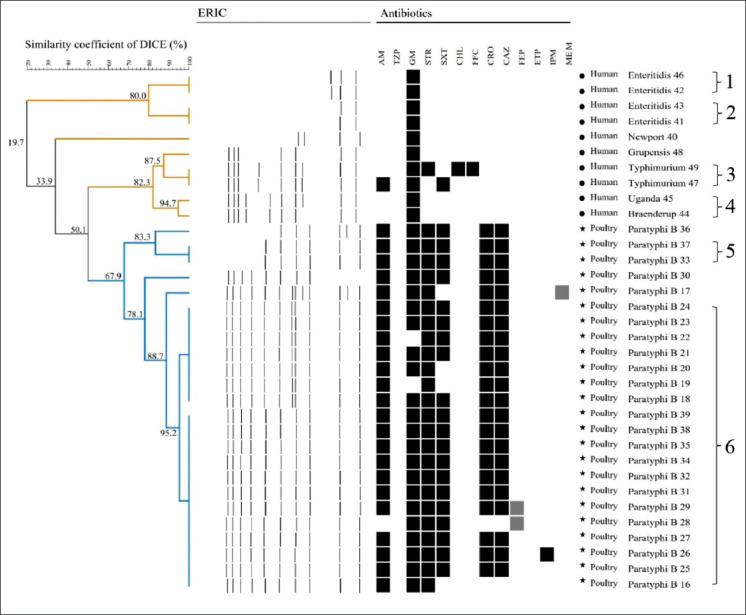
Dendrogram generated from enterobacterial repetitive intergenic consensus-PCR of 34 strains showing source, the serotype of *Salmonella* strains and phenotypic resistance of the strains. AM=Ampicillin, TZP=Piperacillin/tazobactam, GM=Gentamicin, STR=Streptomycin, SXT=Trimethoprim/sulfamethoxazole, CHL=Chloramphenicol, FFC=Florfenicol, CRO=Ceftriaxone, CAZ=Ceftazidime, FEP=Cefepime, ETP=Ertapenem, IPM=Imipenem, MEM=Meropenem, Black=Resistant, White=Susceptible, Gray=Intermediate.

Genotyping of *Salmonella* Heidelberg strains (n=15) isolated from the broiler farms in Santander region using the ERIC primers did not generate band patterns. However, in 34 of the 49 *Salmonella* spp. strains isolated from the broiler farms in Tolima and from human subjects with gastroenteritis, the molecular typing method ERIC generated 2-13 bands ranging in size from 200 to 4000 bp. The dendrogram revealed that the ERIC-PCR method could discriminate the strains according to their source of isolation from the broiler farms in Tolima and from human subjects with gastroenteritis. This typing method grouped the strains into six clusters at a Dice coefficient similarity of 90%, with a discriminatory index of 0.69 (Figure-1). The majority of isolates of the serotype *S*. Paratyphi B were grouped into cluster 6, and the four strains of the serotype *S*. Enteritidis were grouped into two clusters 1 and 2 (Figure-1). Furthermore, the strains grouped in each cluster had variable antibiotic resistance profiles.

## Discussion

In the present study, the strains corresponding to the isolates from broiler farms in Santander and Tolima regions were serotyped as *Salmonella* Heidelberg (n=15) and *Salmonella* Paratyphi B (n=24), respectively. *S*. Heidelberg represents a significant concern as it has been associated with food-borne infections in humans due to the consumption of poultry products [17]. Moreover, a high prevalence in chicken meat may indicate the risk associated with this product as a potential vehicle for the transmission of this bacterium [18]. The strains isolated from human subjects with gastroenteritis corresponded generally to *Salmonella* Enteritidis and *Salmonella* Typhimurium, which are the major serotypes that cause diseases in humans and have been reported previously from clinical cases in Colombia during the period 2005-2011 [19,20].

The results of the phenotypic resistance indicated that the *Salmonella* spp. strains isolated from broiler farms in Santander and Tolima could be categorized as resistant to MDR, that is, bacteria exhibiting resistance to one or more antibiotics from three or more classes of antibiotics [21]. These bacteria were resistant to β-lactams, aminoglycosides, and cephalosporins. The previous studies have reported that *Salmonella* spp. strains isolated from animal products were MDR strains in Colombia [22] and Brazil [23]. In the present study, we detected a high percentage of *Salmonella* strains that were phenotypically resistant to STR (79.5%); these findings are consistent with the previous studies that reported that strains isolated from broiler farms in Cundinamarca (Colombia) [22] and in the United States [24] exhibited a high percentage of resistance to STR. Regarding the resistance to AM (79.5%), previously studies from Santander [22] and Brazil [25] have reported high resistance rates. Moreover, 75.5% of the strains were found to be resistant to CRO and CAZ, which are higher than the results reported from Cuba (CRO, 10.7%; CAZ, 17.9%) [26], Cundinamarca (CRO, 0%; CAZ, 18.2%), and Santander (CRO, 4.5%; CAZ, 69.7%) in Colombia [22]. Resistance to third-generation cephalosporins exhibited by the strains isolated from broiler farms represents a concern because CRO and CAZ are the antibiotics used for salmonellosis treatment in adults and specifically in children, thus rendering the transmission of resistant bacteria a public health problem [26].

The strains isolated from human subjects with gastroenteritis were resistant to GM, which is one of the major antibiotics used in the treatment of urinary infections in humans [27]. On the other hand, all the *Salmonella* spp. strains were susceptible to ETP, which is similar to the result reported by Donado *et al*. [22]. Carbapenems are the final choice of antibiotics used in the treatment of salmonellosis when the bacteria exhibit resistance to antibiotics such as ciprofloxacin and CRO [28].

In the present study, the genotypic analyses revealed the presence of the gene *bla*_CMY2_ in all the strains. This gene encodes an extended-spectrum beta-lactamase that is responsible for hydrolyzing the β-lactam ring preventing it from combining with the penicillin-binding protein (PBP) [29]. This gene confers resistance to AM, ceftiofur, and cefoxitin and is associated with mobile elements, thereby increasing the probability of transmission between bacteria [30]. In our study, 69.4% of the strains demonstrated the presence of the genes *bla*_PSE-1_ and *bla*_TEM_ that encode beta-lactamases that confer resistance to AM. In a study conducted in Ibagué (Colombia), 100% of the strains isolated from chicken carcasses had the gene *bla*_TEM_ [31], a frequency that was higher than that found in the present study. Some strains that were phenotypically resistant to AM and CRO showed the presence of the genes *bla*_PSE-1_, *bla*_TEM_, *bla*_CMY2_, and *bla*_CTX-M_. However, in the case of *Salmonella* Heidelberg isolates, these strains were found to be phenotypically resistant to AM, but none of the genes evaluated in this study were found. It is possible that this resistance is mediated by other mechanisms, such as alterations in the target sites of beta-lactams (PBP) [32] or excessive expression of efflux pumps [33]. Regarding the strains isolated from human subjects and broiler farms in Tolima that had the gene but were phenotypically susceptible; Álvarez [34] mentioned that the hydrolysis generated by these ESBLs in the antibiotic differs between strains, although there is some hydrolysis performed by these enzymes, this is not sufficient to provide clinical resistance in the bacteria [35]. In addition, the gene *sul2* was found in 93.9% of the strains; this gene encodes DHPS (dihydropteroate synthase) that has no affinity for the antibiotic [36]. Different results were found in strains isolated from chicken carcasses in Portugal (37%) [37]. In a previous study, the gene *sul1* was reported to be the most prevalent [37], whereas in the present study, it was found in 57.1% of the strains. In contrast, trimethoprim resistance is mediated by the expression of the enzyme DHFR (dihydrofolate reductase) that has no affinity for the antibiotic and is encoded by the *dfrA1* gene [38] that was detected in 69.4% of the strains. In general, the strains that were resistant to SXT showed the *sul* and/or *dfrA* resistance genes. However, in the case of strains isolated from human subjects, the *sul2*, *dfrA1*, and *dfrA12* genes were present, but they were phenotypically susceptible, which may be due to the inactivation of these genes, as previously reported [39].

The STR resistance gene *aadA1* was detected in 87.8% of the strains; this gene encodes a nucleotidyltransferase that catalyzes the transfer of a nucleotide monophosphate to a hydroxyl group in STR [40]. This result is higher than those reported in *Salmonella* strains isolated in Iran (45.6%) [41] and Thailand (17%) [42]. On the other hand, the gene *strB* also confers resistance to STR and encodes a phosphotransferase that catalyzes the ATP-dependent phosphorylation of hydroxyl groups in STR [43]. The *strB* gene was found in 87.8% of the strains, and similar results have been reported in *Salmonella* strains isolated from chicken carcasses in Ibagué [31]. Regarding the strains isolated from human subjects, it was observed that the strains contained three of four genes that conferred resistance to STR, whereas some strains were not resistant to this antibiotic. In a previous research, White *et al*. [44] reported *Salmonella* strains isolated from meat that had the *aadA2* gene but were susceptible to STR, as the gene was silenced.

The *aadB* gene that confers resistance to GM was not found in any of the strains in the present study. However, the strains isolated from human subjects were resistant to this antibiotic. This resistance may be mediated by other resistance genes such as *grm* [45], which was not evaluated in this study. In contrast, the *S*. Typhimurium serotype isolated from human subjects that were phenotypically resistant to amphenicols did not present the genes *catA* and *cmlA*, which confer resistance to these antibiotics. However, this strain had the *floR* gene, which might explain the resistance of this strain. This gene encodes an efflux pump that confers resistance to amphenicols, which has been reported in the genomic island of *Salmonella* (SGI1) [46,47]. Earlier, this gene was reported in 90% of chloramphenicol-resistant strains isolated from clinical cases in Saudi Arabia [48].

The genotyping method ERIC-PCR is a technique that allows the typing of different *Salmonella* serotypes such as Typhimurium, Paratyphi B, Heidelberg, Pullorum, and Gallinarum [9,49-51]. However, in the present study, we could not type the *Salmonella* Heidelberg strains isolated from broiler farms in Santander using the ERIC-PCR method. Previously, Wilson and Sharp [52] described that mutations in the ERIC region as well as deletions of 60-75 bp can interfere with the site of primer annealing and hinder the generation of band patterns.

This molecular typing method generated 2-13 bands ranging in size from 200 to 4000 bp, which is different from the results reported by Fendri *et al*. [53] (150-1500 bp) and Oliveira *et al*. [7] (190-1430 bp). Moreover, the discriminatory index of this molecular typing method was 0.69, which is similar to that reported by Nath *et al*. [54] who found a discrimination index of 0.70 but lower than those reported by Fendri *et al*. [53] and Kim and Lee [55] who found discriminatory indexes of 0.85 and 0.97, respectively. However, in the present study, the ERIC-PCR method was able to discriminate the strains according to their source of isolation, and a greater discriminatory power does not always represent a grouping with biological significance [56].

The serotype Paratyphi B was grouped into cluster 6, but some strains were also not grouped in a cluster; these results are in agreement with those reported by Kim and Lee [55] who described that ERIC-PCR can be used to sub-typify within the serotype. In addition, *S*. Enteritidis strains were grouped into two clusters; this is possible due to the fact that *S*. Enteritidis strains could have divergent clonal lineages so that they can be grouped into a different cluster [49]. Finally, in the present study, no relationship was found between the clusters and the antibiotic resistance. Kim and Lee [55] and Oliveira *et al*. [7] also reported similar results in which the clusters did not exhibit the same pattern of antibiotic resistance.

## Conclusion

*Salmonella* spp. isolated from broiler farms in Santander and Tolima were MDR strains and demonstrated the presence of resistance genes associated with such resistance. Furthermore, ERIC-PCR is a technique that allowed obtaining clusters with biological significance, although this genotyping method could not type the Heidelberg strains.

## Authors’ Contributions

ISR and MPH were responsible for the design of the study. MPH performed the experiments and laboratory analyses. ISR administered the project. RR collected the strains. ISR and MPH wrote the manuscript. ISR, MPH and RR reviewed and edited the paper. ISRB revised the manuscript critically. All authors read and approved the final manuscript.
